# The Cellular Prion Protein: A Promising Therapeutic Target for Cancer

**DOI:** 10.3390/ijms21239208

**Published:** 2020-12-02

**Authors:** Gyeongyun Go, Sang Hun Lee

**Affiliations:** 1 Department of Biochemistry, Soonchunhyang University College of Medicine, Cheonan 31151, Korea; ggy0227@naver.com; 2 Medical Science Research Institute, Soonchunhyang University Seoul Hospital, Seoul 04401, Korea

**Keywords:** cellular prion protein, PrP^C^, *PRNP*, cancer, cancer stem cell, targeted cancer therapy

## Abstract

Studies on the cellular prion protein (PrP^C^) have been actively conducted because misfolded PrP^C^ is known to cause transmissible spongiform encephalopathies or prion disease. PrP^C^ is a glycophosphatidylinositol-anchored cell surface glycoprotein that has been reported to affect several cellular functions such as stress protection, cellular differentiation, mitochondrial homeostasis, circadian rhythm, myelin homeostasis, and immune modulation. Recently, it has also been reported that PrP^C^ mediates tumor progression by enhancing the proliferation, metastasis, and drug resistance of cancer cells. In addition, PrP^C^ regulates cancer stem cell properties by interacting with cancer stem cell marker proteins. In this review, we summarize how PrP^C^ promotes tumor progression in terms of proliferation, metastasis, drug resistance, and cancer stem cell properties. In addition, we discuss strategies to treat tumors by modulating the function and expression of PrP^C^ via the regulation of HSPA1L/HIF-1α expression and using an anti-prion antibody.

## 1. Introduction

The cellular prion protein (PrP^C^) is a cell surface glycophosphatidylinositol (GPI)-anchored protein consisting of 208 amino acids, and it is encoded by the *PRNP* gene located on chromosome 20. PrP^C^ has been intensively studied since it was proposed that misfolding of PrP^C^ plays a key role in the pathogenesis of neurodegenerative diseases called transmissible spongiform encephalopathies [1,2,3]. Studies have shown that PrP^C^ is not simply a cause of neurodegenerative diseases, but it is an important protein involved in many cellular functions such as stress protection, cellular differentiation, mitochondrial homeostasis, circadian rhythm, myelin homeostasis, and immune modulation [4,5,6,7,8,9,10]. Furthermore, several studies have shown that PrP^C^ expression is associated with tumor progression [11,12,13,14,15]. Before addressing the role of PrP^C^ in tumor progression, we briefly introduce herein some biochemical aspects of PrP^C^.

PrP^C^ is first synthesized as a precursor protein (pre-pro-PrP) comprising 253 amino acids with a signal peptide at the N-terminus and a GPI anchor peptide signaling sequence (GPI-PSS) at the C-terminus. The signal peptide directs pre-pro-PrP into the endoplasmic reticulum (ER), wherein it is cleaved to generate pro-PrP. The pro-PrP is then translocated from the ER to the Golgi complex [16,17] to be further processed by the addition of N-linked glycans, removal of the GPI-PSS, and addition of the pre-assembled GPI anchor [18,19]. Finally, the mature PrP^C^ of 208 amino acids is translocated to the outer membrane leaflet of cells. However, not all PrP^C^s are present on the cell surface. They are constantly internalized through the recycling endosome and trafficked back repeatedly [20,21,22]. Through this recycling process, PrP^C^s are also found in the Golgi [22,23], in addition to the nucleus [24,25] and mitochondria [26,27].

The relationship between PrP^C^ and cancer progression was first discovered when *PRNP* was identified as one of the most-expressed genes in pancreatic cancer cells [28]. Around the same time, other researchers found that PrP^C^ was overexpressed in a drug-resistant cancer cell line compared to the parental cell line [29]. Based on several studies, it is now well established that PrP^C^ is involved in the main aspects of cancer biology: proliferation, metastasis, and drug resistance. Moreover, the relationship between PrP^C^ and cancer stem cell phenotypes has also been uncovered [30,31]. In this review, we summarize the role of PrP^C^ in tumor progression in terms of proliferation, metastasis, drug resistance, and cancer stem cell properties. Finally, we discuss strategies to control tumor growth by regulating the function and expression of PrP^C^.

## 2. Overview of Physiological Functions of PrP^C^

PrP^C^ is known to regulate several functions of cells, such as stress protection, cellular differentiation, mitochondrial homeostasis, circadian rhythm, myelin homeostasis, and immune modulation. In this review, we briefly summarize the effects of PrP^C^ on stress protection, cellular differentiation, and mitochondrial homeostasis.

Several studies have shown that PrP^C^ can directly inhibit apoptosis. PrP^C^ expression inhibited mitochondria-dependent apoptosis in Bax-overexpressing human primary neurons and MCF-7 breast cancer cells [32,33]. In addition, downregulation of PrP^C^ reduced the viability of MDA-MB-435 breast cancer cells after serum deprivation [34]. In primary hippocampal neurons, PrP^C^ protected the cells against staurosporine-induced cell death by interacting with stress-induced phosphoprotein 1 (STI1) [35,36,37]. PrP^C^ is also known to protect cells from oxidative stress. For example, the basal levels of ROS and lipid peroxidation were lower in PrP^C^-transfected neuroblastoma and epithelial cell lines than in untransfected controls [38,39]. In addition, the expression of PrP^C^ by primary neurons and astrocytes has been associated with lower levels of damage caused by the addition of various oxidative toxins such as xanthine oxidase, kainic acid, and hydrogen peroxide [40,41]. PrP^C^ has also been found to be involved in the ER-stress response. When breast carcinoma cells were treated with the ER-stress inducing compounds such as brefeldin A, tunicamycin, and thapsigargin, the expression of PrP^C^ was induced. Downregulation of PrP^C^ in several cancer cell lines resulted in an increase in cell death in response to these toxins [13].

Neurite outgrowth is one of the characteristics of neuronal differentiation. Several studies have indicated that PrP^C^ promotes neurite outgrowth through interactions with other proteins such as neural cell adhesion molecule 1, epidermal growth factor receptor, integrins, laminin, and STI1 [35,42,43,44,45]. The downstream signaling of these interactions may include RhoA-Rho kinase-LIMK-cofilin pathway [44]. Activation of various signal pathways, including extracellular signal-regulated kinases 1 and 2 (ERK1/2), phosphatidylinositol-3-kinase (PI3K)/Akt, and mitogen-activated protein kinases (MAPKs), may also induce PrP^C^-dependent neurite outgrowth [35,43,46]. It has been reported that PrP^C^ is also involved in the differentiation of embryonic stem cells. In human embryonic stem cells, downregulation of PrP^C^ delays spontaneous differentiation into the three germ layers [47]. Similarly, PrP^C^ expression promotes the differentiation of cultured human embryonic stem cells and multipotent neural precursors to mature neurons, astroglia, and oligodendroglia [47,48].

PrP^C^ expression also affects mitochondrial homeostasis. Transcriptomic and proteomic analyses of brain tissues and neurons of PrP^C^-null and wild-type mice have identified differently expressed proteins. These proteins include cytochrome c oxidase subunits 1 and 2, which are involved in oxidative phosphorylation [49,50]. Furthermore, the absence of PrP^C^ reduces the number of total mitochondria and increases the number of mitochondria with unusual morphology [49].

## 3. PrP^C^ and Cancer Proliferation

PrP^C^ expression has been reported to promote cancer proliferation in several types of cancer cells, including gastric [51], pancreatic [52], and colon [53,54,55], as well as in glioblastoma (GBM) [56,57] and schwannanoma [58].

In gastric cancer, PrP^C^ promotes cell proliferation and metastasis of cancer cells and promotes tumor growth in xenograft mouse models [51]. PrP^C^ increases the expression of cyclin D1 and thereby promotes their transition from the G0/G1 phase to the S-phase. PrP^C^ expression also affects Akt signaling. Overexpression of PrP^C^ increases p-Akt levels, whereas PrP^C^ knockdown inhibits p-Akt expression [59]. Interestingly, it is known that certain regions of PrP^C^ influence cell proliferation. Specifically, deletion of amino acids 24–50 of PrP^C^ significantly reduced cell proliferation. Conversely, deletion of amino acids 51–91 did not affect apoptosis, metastasis cell proliferation, and multidrug resistance in gastric cancer [60].

In pancreatic ductal adenocarcinoma (PDAC), expression of PrP^C^ increases the proliferation and migration of the cells. In PDAC cell lines, PrP^C^ exists as a pro-PrP as it retains its GPI-PSS, which has a filamin A (FLNA) binding motif. It was found that the interaction between pro-PrP and FLNA, a cytoplasmic protein involved in actin organization, promotes cell migration [61]. In addition, other studies have shown that PrP^C^ promote pancreatic cell proliferation by activating the Notch signaling pathway [62].

PrP^C^ is known to interact with other membrane proteins or extracellular molecules to perform various cellular functions. In human GBM, PrP^C^ and heat shock 90/70 organizing protein (HOP) are upregulated, and their expression levels correlate with higher proliferation rates and poorer clinical outcomes [56]. Additionally, it has been demonstrated that the binding of HOP to PrP^C^ promotes proliferation of GBM cell lines and that disruption of PrP^C^–HOP interaction inhibits tumor growth and improves the survival of mice [56].

In DLS-1 and SW480 colorectal cancer cells, knockdown of PrP^C^ significantly reduces the proliferation of cancer cells. It is known that the binding between HIF-2α and the GLUT1 promoter region decreases when PrP^C^ expression is suppressed, resulting in a decrease in the expression of GLUT1. This may reduce glucose uptake and glycolysis and inhibit cell proliferation [54].

## 4. PrP^C^ and Metastasis

It has been demonstrated that PrP^C^ promotes the invasion and migration of several types of cancer cells, such as gastric [63], pancreatic [62], colon [64], and melanoma [65] cells.

PrP^C^ expression is higher in metastatic gastric cancer than in non-metastatic gastric cancer. PrP^C^ increases the invasion and in vivo metastatic ability of gastric cancer cell lines SGC7901 and MKN45, and knockdown of PrP^C^ significantly reduces cancer cell invasion [63]. PrP^C^ seems to induce cancer cell invasion by activating the p-ERK1/2 signal and inducing the expression of MMP11. Interestingly, the N-terminal fragment (amino acids 24–90) of PrP^C^ has been proposed as the region for its invasion-promoting function.

PrP^C^ levels were found to increase in invasive melanoma, whereas in normal melanocytes, PrP^C^ was not detected [65]. In melanoma, PrP^C^ is known to exist as pro-PrP, retaining its GPI-PSS with an FLNA binding motif. As in PDAC earlier, PrP^C^ promoted migration by binding with FLNA and regulating cytoskeleton organization. PrP^C^ knockdown significantly reduced the migration of melanoma cells in a wound healing assay [65,66].

In colon cancer, PrP^C^ is known to promote migration by binding to HOP, also known as stress-induced phosphoprotein 1 (STI1) [64]. It was found that among the colon primary tumor cells, only PrP^C^-positive cells were able to promote liver metastasis after injection into immunocompromised mice [67]. Metastasis is highly correlated with epithelial-mesenchymal transition (EMT), a process in which cells lose epithelial markers and interaction between the cell and the extracellular matrix change the cytoskeleton organization and differentiate into mesenchymal phenotype [68]. It is well known that transcription factors such as SNAI1, SNAI2, TWIST1, TWIST2, ZEB1, and ZEB2 induce EMT. *PRNP* expression is highly associated with the EMT signature in colon cancer patients, and PrP^C^ is known to control the expression of ZEB1 in colon cancer cells [53].

## 5. PrP^C^ and Drug Resistance

PrP^C^ levels were found to be higher in tumor necrosis factor α (TNF-α)-resistant breast cancer cells than in TNF-α-sensitive breast cancer cells [33]. After treatment of TNF-α, the resistant cells did not exhibit cytochrome c release or nuclear condensation. Moreover, PrP^C^ expression inhibited the Bax translocation to mitochondria and Bax-mediated cytochrome c release. In addition to TNF-α, PrP^C^ is also involved in the resistance to adriamycin (ADR) and TRAIL-mediated cell death in breast cancer cells as down-regulation of PrP^C^ increased the sensitivity to these molecules. [69]. Inhibition of PrP^C^ expression did not inhibit formation of death-inducing signaling complex (DISC); however, it inhibited Bcl-2 expression and promoted Bid cleavage, resulting in cell death [69]. In addition, it has been confirmed that PrP^C^ co-localization and coexpression with p-glycoprotein (P-gp) occur in ADR-resistant MCF-7 cells. When the expression of PrC^C^ was inhibited in these cells, the capability of paclitaxel, a P-gp substrate, to induce in vitro invasion of the cells decreased [52]. More importantly, tissue microarray analysis of 756 breast cancer tumors demonstrated that PrP^C^ was associated with ER-negative breast cancer subsets, and compared with ER-negative/PrP^C^-positive cells, ER-negative/PrP^C^-negative cells are more sensitive to adjuvant chemotherapy [70].

PrP^C^ is also involved in drug resistance in colon cancer. Hypoxia-induced PrP^C^ expression in colorectal cancer cells inhibits TRAIL-induced apoptosis [71]. PrP^C^ inhibited apoptosis of colon cancer cells by activating the PI3K-Akt pathway [72]. Conversely, deletion of PrP^C^ resulted in reduced Akt activation and enhanced caspase-3 activation [73]. Our group has demonstrated that PrP^C^ levels significantly increase in 5-FU-and oxaliplatin-resistant colorectal cancer [74,75,76,77]. In addition, knocking down PrP^C^ expression significantly reduces the drug resistance of colorectal cancer cells. Furthermore, we have shown that PrP^C^ suppresses the drug-induced activation of stress-associated proteins, such as p38, JNK, and p53. We have also demonstrated that PrP^C^ inhibits caspase-3 activation by drugs and PARP1 cleavage. These results suggest that the level of PrP^C^ plays an important role in the development of drug resistance in colorectal cancer cells.

## 6. PrP^C^ and Cancer Stem Cells

Cancer stem cells (CSCs) are a subpopulation of cells that are capable of self-renewal, differentiation into various cell types in a determined tumor, and tumor propagation when xenotransplanted into mice [78,79]. CSCs are resistant to conventional medical therapies and have been implicated in cancer recurrence, which has made these cells a key target for therapy [80,81,82].

Recently, studies on the correlation between PrP^C^ and CSCs have been conducted. PrP^C^ activation of Fyn-SP1 pathway in colon cancer cells promoted EMT and resulted in a more aggressive phenotype [83]. EMT is closely connected with CSC properties as EMT enhances metastasis and drug resistance of cancer and cancer microenvironment promotes activation of EMT program [84]. Du et al. demonstrated that PrP^C^-positive primary colon cancer cells expressed high levels of the EMT-associated markers, TWIST and N-cadherin, and low levels of the epithelial marker, E-cadherin, as well as exhibiting CSC properties such as the expression of the CSC marker, CD44, and tumor-initiating capacity [67].

In line with this finding, PrP^C^ has been shown to interact with CD44 in multidrug-resistant breast cancer cells [85]. Furthermore, in primary GBM cells, PrP^C^ silencing reduces the expression of the CSC markers, Sox2 and Nanog, as well as the self-renewal and tumorigenic potential of CSCs [57]. Similar findings were demonstrated by Iglesia et al., who worked on GBM cell lines grown as neurospheres [86].

CSCs are one of the most-studied recent topics in cancer biology. They have emerged as pivotal components that can initiate and maintain tumors [79]. PrP^C^ is known to interact with CD44, and its expression correlates with resistance to chemotherapy in breast cancer cell lines [85]. Moreover, the CD44-positive and PrP^C^-positive subpopulations of colorectal tumor cells have CSC properties, including tumorigenic and metastatic capacities [67], indicating that PrP^C^ contributes to tumor maintenance by modulating CSC behaviors.

Our group confirmed the correlation between PrP^C^ and CSC by demonstrating that the levels of PrP^C^ and CSC marker proteins such as Oct4, Nanog, Sox2, and ALDH1A1 significantly increased in human colorectal cancer tissues and colorectal cancer cells [31]. In addition, knockdown of PrP^C^ reduced the expression of CSC markers in the CSCs. More specifically, PrP^C^ inhibited the anticancer drug-induced degradation of Oct4, but did not inhibit the degradation of other stem cell markers such as Nanog, Sox2, and ALDH1A1. Oct4 is a master regulator involved in the self-renewal and pluripotency of CSCs. It has been reported that tumor sphere formation ability is activated in breast cancer cells overexpressing Oct4 [87,88]. Cancer cells overexpressing Oct4 also overexpress other CSC markers such as CD133, CD34, and ALDH1. Oct4 is also involved in the survival, self-renewal, metastasis, and drug resistance of CSCs [89,90,91,92]. These results indicate that PrP^C^ directly regulates Oct4 expression, whereas it indirectly regulates Nanog, Sox2, and ALDH1A to promote the self-renewal and survival of CSCs [31].

**Figure 1:**
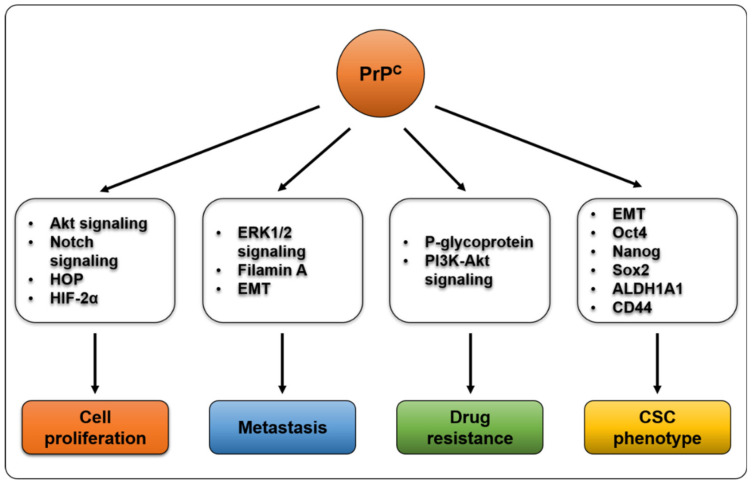
Proteins and signaling pathways that seem to be affected by PrP^C^ expression. Various proteins and signaling pathways reportedly interact with or are modulated by PrP^C^. Some of the proteins shown are known to be regulated indirectly through other proteins rather than direct interaction with PrP^C^. PrP^C^: prion protein, ECM: extracellular matrix, CSC: cancer stem cell.

In summary, Figure 1 shows the proteins and signaling pathways that seem to be affected by PrP^C^ expression. The information on these interactions was retrieved from several studies that have been already mentioned in this review. Although PrP^C^ appear to interact and activate several interaction partners and signaling pathways to promote tumor progression, it is difficult to say that this applies to all cancer cells. The role of PrP^C^ in cancer needs to be interpreted differently depending on the cell type and interaction partner.

## 7. Cancer Treatment by Targeting PrP^C^

Cancer growth can be inhibited by inhibiting the interaction between PrP^C^ and other proteins. Lopes et al. used a peptide named HOP/STI1230–245 corresponding to the prion binding site of HOP to inhibit the interaction between PrP^C^ and HOP [56]. Treatment with only the peptide did not inhibit cell proliferation, but co-treatment with HOP inhibited the interaction between HOP and PrP^C^ and HOP-induced cell proliferation. In addition, HOP/STI1230–245 treatment of orthotopic xenografts inhibited tumor growth and improved animal survival while maintaining cognitive performance [56]. It should be noted that the blockage of PrP^C^ and HOP may be deleterious, because long-term [93] but not short-term [94] intracranial infusion of antibodies against PrP^C^, particularly those against its globular domain, can be neurotoxic [93].

**Figure 2:**
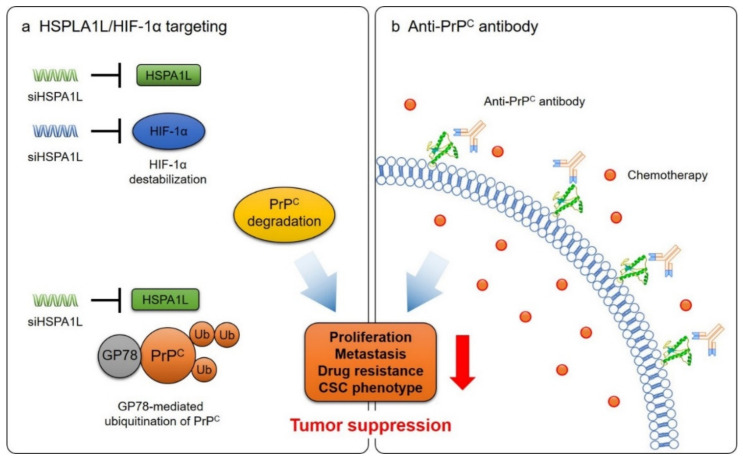
Strategies to suppress the tumor progression by regulating the expression and function of PrP^C^. (**a**) In tumor niche, the expression of HSPA1L is increased. HSPA1L binds and stabilizes HIF-1α. HSPA1L directly binds to GP78 and inhibits its ubiquitination activity. Overall, PrP^C^ expression is increased in tumor cells. Therefore, knocking down HSPA1L and HIF-1α expression induces degradation of PrP^C^ and tumor suppression. (**b**) Direct targeting of PrP^C^ using anti-PrP^C^ antibodies has been demonstrated as a potent cancer therapy. Anti-PrP^C^ antibody also can be used as a combination therapy with conventional anticancer drugs.

Recently, our group identified that HSP family A (Hsp 70) member 1-like, HSPA1L, regulates the expression of PrP^C^ [77]. It was confirmed that the expression of HSPA1L increased in colon cancer cells and cancer tissues. We also demonstrated that HSPA1L increases the stability of HIF-1α by binding with HIF-1α and promotes the accumulation of PrP^C^. When HSPA1L expression was knocked down, HIF-1α stability and PrP^C^ expression decreased [77]. In addition, we also showed that HSPA1L binds to GP78 and inhibits its ubiquitination activity, thereby reducing the ubiquitination of PrP^C^. Several studies have indicated that deregulation of several E3 ligases affects the growth and metastasis of cancer and the growth of CSCs [95,96,97,98]. GP78 is an ER membrane-anchored E3 ligase that regulates the progression of cancer cells through ubiquitin ligase activity. For example, downregulation of GP78-mediated ubiquitination is known to inhibit metastasis in breast cancer cells [99]. We hypothesized that HIF-1α and HSPA1L are major therapeutic targets for colorectal cancer. Indeed, knockdown of HIF-1α and HSPA1L using siRNAs inhibited cancer sphere formation in HT-29 and S707 cells. In an in vivo xenograft model, knockdown of HIF-1α or HSPA1L inhibited tumor growth and liver metastasis (Figure 2). In addition, when both genes were knocked down simultaneously, cancer growth and liver metastasis were further suppressed [77]. These results indicated that PrP^C^ is important in tumor progression, and the suppression of PrP^C^ expression by targeting HIF-1α and HSPA1L could be a promising therapeutic strategy to treat cancer.

Anti-prion antibodies can be utilized for the treatment of cancer. Antibody therapeutics have been used in the treatment of cancer, unlike existing anticancer drugs, as they have fewer side effects and exhibit high efficacy. For the development of effective antibody therapeutics, the discovery of specific molecular biomarkers in a wide range of solid malignancies is a key process [100]. The functions of PrP^C^ in the growth, metastasis, drug resistance, and CSC properties of various types of cancer suggest that it is a promising therapeutic target for cancer treatment. We confirmed that an anti-prion antibody showed anticancer effects in a xenograft model and that superior therapeutic effects appeared when the conventional anticancer drugs and anti-prion antibody were applied in combination (unpublished data) (Figure 2). Furthermore, compared to cetuximab, an EGFR-targeting antibody, the anti-prion antibody showed similar anticancer effects with 10 times lower dose in the xenograft mouse model (unpublished data).

Similar to our data, a previous study has revealed the effective epitope of PrP^C^ for antibody-mediated colon cancer therapy [101]. In colon cancer cell line HCT116 cells, epitope 139–142 and epitope 141–151 targeting anti-PrP antibodies highly inhibited the proliferative capacity of cells, compared with those of epitope 93–109 and epitope 101–112 [101]. Furthermore, epitope 141–151 targeting anti-PrP was approximately 10-fold more active than that of epitope 93–109 targeting anti-PrP [101]. These data indicate that effectiveness of anti-PrP antibodies might be related to the epitope-binding region. Further studies on anti-PrP structure and its targeting epitope site for cancer therapy are needed. One study has shown that N-terminal domain of PrP^C^ is a direct binding and sequestering site on anti-tumor drug, doxorubicin, in breast cancer [67]. This study suggests that N-terminal-domain-targeting anti-PrP antibodies might be effective antibody therapy when combined with anti-tumor drugs, for cancer treatment.

Although antibody therapy is a promising cancer treatment, resistance may arise due to the characteristics of cancer cells, such as intrinsic phenotypic variation and adaptive phenotypic modifications [100,102,103,104,105]. Antibody-drug conjugates (ADCs), which are novel antibody-based therapeutics, are another option for treating tumors. ADC is a technology that focuses on targeting only cancer cells by exploiting the advantages of antibodies: specificity, non-toxicity in circulation, and pharmacokinetics. ADC is known to enter cells through clathrin-mediated endocytosis [106]. The endosome that harbors ADC binds to other vesicles in the cell and forms an endo-lysosome. A protease cleaves the linker of the ADC and activates free drugs to move into the cytoplasm. The drugs bind to the molecular target, causing apoptosis of the tumor cells. A representative example of a successful ADC is Trastuzumab Emtansine (T-DM1) [107,108,109]. T-DM1 significantly prolonged progression-free and overall survival with less toxicity than lapatinib plus capecitabine in patients with HER2-positive advanced breast cancer who were previously treated with trastuzumab and a taxane. Anti-prion antibodies, such as T-DM1, are expected to be developed as anticancer agents in the form of ADCs.

## 8. Conclusions

Several studies have suggested that PrP^C^ promotes tumor progression. It has been demonstrated that PrP^C^ is overexpressed in various types of cancer cells and tumor tissues, including gastric, pancreatic, breast, and colon cancers, as well as melanoma, GBM, and schwannoma. In addition, it has been shown that PrP^C^ regulates cell proliferation, metastasis, drug resistance, and cancer stem cell properties through signaling pathways, such as PI3K-Akt and Notch, and interaction with ECM, cell surface molecules, and cancer stem cell markers. It should be noted that the function of PrP^C^ in cancer should be interpreted depending on the cell type and the molecule that interacts with it. Nevertheless, further research is needed to elucidate the function of PrP^C^ in tumor progression. However, there seems to be no disagreement that PrP^C^ is a promising target for cancer treatment.

In addition to PrP^C^, the misfolded prion protein (PrP^Sc^) may be highly expressed in cancer patients compared to the healthy people. Recently, somatic mutations in *PRNP* were analyzed in 10,967 cancer patients using the Cancer Genome atlas (TCGA) database [110]. A total of 48 mutations in *PRNP* gene were identified in cancer patient. Among them, eight somatic mutations— G131V, D167N, V180I, D202N, V203I, R208C, R208H, and E211Q—are known as pathogenic mutations of prion diseases. Interestingly, it has been reported that PrP^Sc^ was also detected in healthy people, who had not been diagnosed with prion diseases [111]. These results may indicate that cancer patients carrying pathogenic somatic mutations of *PRNP* may produce PrP^Sc^ and may not be diagnosed with prion disease.

In this review, we suggest that prion targeting is a promising strategy to treat cancer. Regulating the expression of HSPL1A and HIF-1α, which are involved in the stability and degradation of PrP^C^, effectively inhibits cancer growth and metastasis. In addition, an anti-prion antibody has been used to inhibit the growth of cancer. Nevertheless, further studies are needed to verify the anticancer effect and safety of prion targeting in various cancer models. To date, no clinical trials using prion targeting have been conducted; therefore, the effectiveness and safety of cancer treatment strategies using prion targeting should also be verified.
